# Considerations for routinely testing for high lipoprotein(a)

**DOI:** 10.1097/MOL.0000000000000838

**Published:** 2022-10-18

**Authors:** Nick S Nurmohamed, Patrick M Moriarty, Erik SG Stroes

**Affiliations:** a Department of Vascular Medicine, Amsterdam UMC, University of Amsterdam, The Netherlands; b Department of Cardiology, Amsterdam UMC, Vrije Universiteit, Amsterdam, The Netherlands; c Atherosclerosis and Lipid-apheresis Center, University of Kansas Medical Center, Kansas City, KS, USA

**Keywords:** atherosclerotic cardiovascular disease risk, guidelines, lipoprotein(a)

## Abstract

Lipoprotein (a) [Lp(a)] is a likely causal risk factor for atherosclerotic cardiovascular disease (ASCVD) and aortic valve disease, confirmed by Mendelian randomization. With reliable assays, it has been established that Lp(a) is linearly associated with ASCVD. Current low-density lipoprotein cholesterol (LDL-C) lowering therapies do not or minimally lower Lp(a). This review focuses on the clinical importance and therapeutic consequences of Lp(a) measurement.

**Recent findings:**

Development of RNA-based Lp(a) lowering therapeutics has positioned Lp(a) as one of the principal residual risk factors to target in the battle against lipid-driven ASCVD risk. Pelacarsen, which is a liver-specific antisense oligonucleotide, has shown Lp(a) reductions up to 90% and its phase 3 trial is currently underway. Olpasiran is a small interfering RNA targeting *LPA* messenger RNA, which is being investigated in phase 2 and has already shown dose-dependent Lp(a) reductions up to 90%.

**Summary:**

Lp(a) should be measured in every patient at least once to identify patients with very high Lp(a) levels. These patients could benefit from Lp(a) lowering therapies when approved. In the meantime, therapy in high Lp(a) patients should focus on further reducing LDL-C and other ASCVD risk factors.

## INTRODUCTION

Lipoprotein(a) [Lp(a)] is a low-density lipoprotein (LDL) particle covalently bound to apolipoprotein(a) and is a likely causal risk factor for atherosclerotic cardiovascular disease (ASCVD) and aortic valve disease [1–4]. Evidence from both observational and Mendelian randomization studies has established Lp(a) as an important ASCVD risk factor [5]. Following improvements in assay technology the last decade, and with the recent introduction of isoform-independent assays, Lp(a) can now be measured reliably. Simultaneously, development of RNA-based Lp(a) lowering therapeutics has positioned Lp(a) as one of the principal residual risk factors to target in the battle against lipid-driven ASCVD risk. This review focuses on these developments and the clinical consequences of – as well as the need for – widespread measurement of Lp(a). 

**Box 1:**
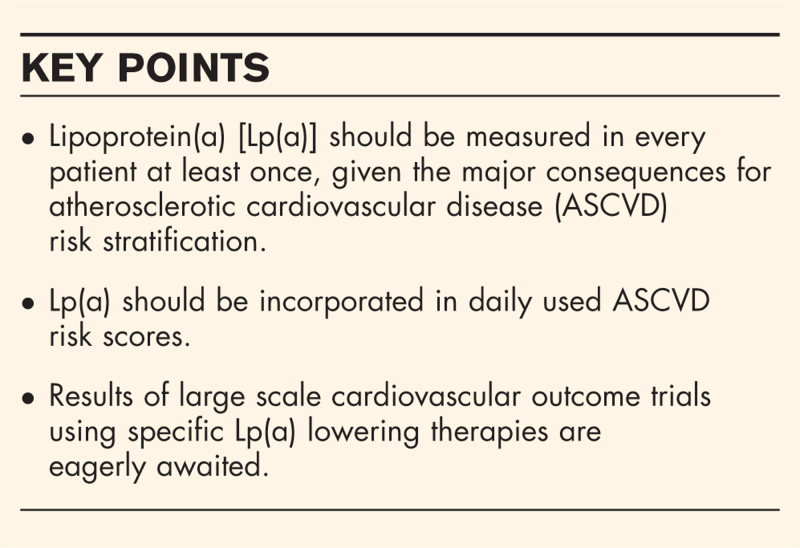
no caption available

## GENETICS, PATHOPHYSIOLOGY AND EPIDEMIOLOGY OF HIGH LIPOPROTEIN(a)

Lp(a) plasma levels are primarily determined by the *LPA* gene on chromosome 6q26–27 [6]. It consists of an LDL-like particle with the addition of an apolipoprotein(a) molecule linked to the apoB-100 protein on LDL by disulphide bonds. Apolipoprotein(a) consists of a protease domain, 10 different kringle IV structures and one kringle V structure. The size of the apolipoprotein(a) tail is determined by the number of kringle IV type 2 repeats, which can vary from 11 to >50 copies [7]. The plasma level of Lp(a) is inversely dependent on the size of the particle [8]. This has hampered reliable estimation of Lp(a) concentration in the past, but the introduction of calibrated mass assays removed this obstacle. Nevertheless, although most mass assays are calibrated to cope with different apo(a) sizes, inter-assay variation hampers one-to-one comparison between hospitals and countries. Most recently, an isoform-independent molar assay was introduced, providing a significant advantage over traditional mass assays, since there is no influence of particle size [9^▪^]. This assay will likely be the gold standard for Lp(a) measurement in the near future.

**FIGURE 1:**
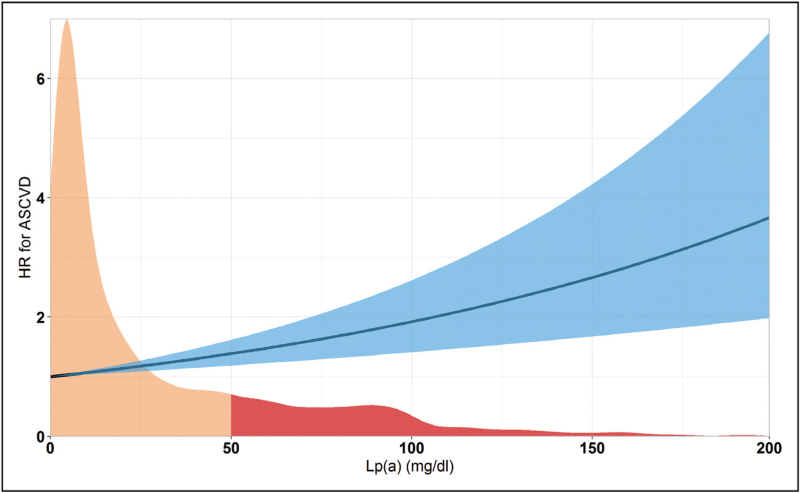
Lp(a) distribution and hazard ratio for myocardial infarction. The distribution of Lp(a) levels in a tertiary hospital population (*n* = 12 437 [32],) is shown in orange and red. Marked in red are individuals with an Lp(a) above the 80th percentile (50 mg/dl). The line represents the hazard ratio for myocardial infarction with the 95% CI in blue. CI, confidence interval; Lp(a), lipoprotein(a).

In the general population, Lp(a) has a distribution skewed to the right and it is estimated that 20% of individuals worldwide has an Lp(a) level above 50 mg/dl or 105 nmol/l (Fig. 1) [10^▪^]. Five percent of individuals has an Lp(a) level above 120 mg/dl or 250 nmol/l, whereas 1% of individuals has an extremely elevated Lp(a) level above the ^99th^ percentile, which corresponds to approximately 180 mg/dl [10^▪^]. Plasma levels of Lp(a) are also dependent on ethnicity: individuals from African descent generally have higher Lp(a) levels, whereas plasma Lp(a) levels are generally lower in Asian individuals, when compared with Caucasian individuals [11]. The clinical implications of these differences in Lp(a) distribution for ASCVD risk stratification still remain to be determined [12].

**FIGURE 2:**
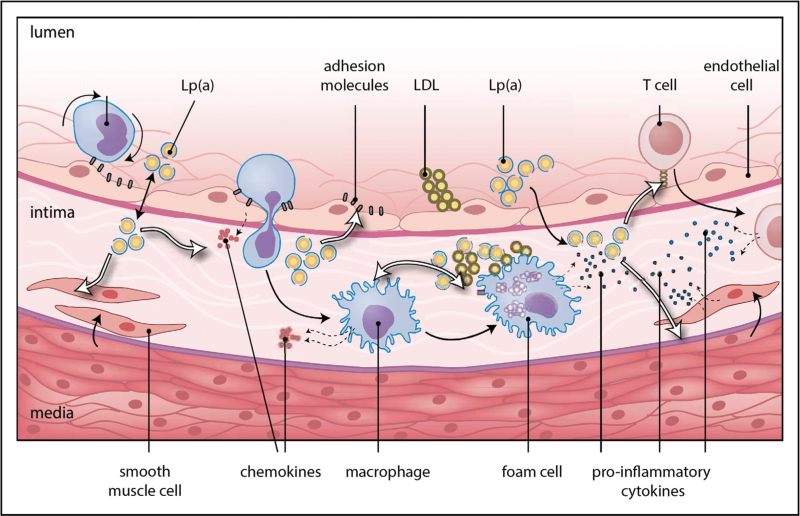
High lipoprotein(a) results in atherosclerosis. Simplified pathophysiology of Lp(a) in the arterial wall. The potential antifibrinolytic properties of Lp(a) due to its structural similarity to plasminogen are not shown in this figure. LDL, low-density lipoprotein; Lp(a), lipoprotein(a). Adapted and reproduced with permission from Nurmohamed *et al.* Focus Vasculair 2021, Prelum.

Lp(a) contributes to atherosclerosis and ASCVD risk through multiple mechanisms (Fig. 2). Lp(a), as a particle consisting of an LDL core with an apo(a) part, contains cholesterol similar to LDL particles, and therefore carries the risk associated with LDL particles. In addition, the apo(a) part of the particle carries oxidized phospholipids, which are recognized as damage-associated molecular patterns by receptors on innate immune cells and have other pro-atherosclerotic and pro-inflammatory effects [13]. The apo(a) tail, due to its structural homology to plasminogen but without its enzymatic function, has also been suggested to possess antifibrinolytic properties [8].

Mendelian randomization and observational studies have shown that Lp(a), which level is fairly constant throughout an individual's lifetime, is at least linearly related to ASCVD risk. In a Mendelian randomization analysis, every 10 mg/dl increase in Lp(a) above the population median was associated with a 5.8% increased risk for coronary artery disease (Fig. 1) [5]. In the Copenhagen City Heart Study, it was observed that individuals with an Lp(a) above the 80th percentile had a hazard ratio for myocardial infarction of 2.0 (95% confidence interval [CI] 1.6–4.1) [14], which further increases with higher Lp(a) levels to 2.6 (95% CI 1.6–4.1) for patients above the 95th percentile [2]. For ischemic stroke, the hazard ratio was 1.6 (95% CI 1.2–2.1) [15]. Hazard ratios for cardiovascular and all-cause mortality were 1.5 (95% CI 1.3–1.8) and 1.2 (95% CI 1.1–1.3) for individuals with an Lp(a) above the 95th percentile from two Copenhagen cohorts [16].

In addition to its contribution to ASCVD risk, high Lp(a) drives calcification of the aortic valve, leading to aortic valve stenosis. Lp(a) is thought to be primarily important for the initiation phase of aortic valve stenosis [17]. Exposure of the aortic valve to Lp(a) eventually leads to endothelial damage and infiltration of Lp(a), after which release of inflammatory mediators stimulates valvular interstitial cells into apoptosis and osteoblastic differentiation [17]. As a result, the calcium depositions by the osteoblast initiate the propagation phase where increased mechanical stress and injury result in further disease progression. Mendelian randomization studies estimated a 62% increase of aortic valve stenosis risk for each 10-fold Lp(a) increase. Individuals with Lp(a) levels above the 95th percentile had a hazard ratio of 2.9 (95% CI 1.8–4.9) for calcific aortic valve stenosis in two Copenhagen cohorts (*n* = 77 680) [4,18].

## PHARMACOLOGICAL LOWERING OF HIGH LIPOPROTEIN(a)

### Statins, ezetimibe and proprotein convertase subtilisin kexin type 9 inhibition

Statins and ezetimibe, which form the current starting point of low-density lipoprotein cholesterol (LDL-C) lowering, do not sufficiently lower plasma Lp(a). In fact, in a meta-analysis of 6 RCTs, statins even increase plasma Lp(a) levels up to 10–20% [19], whereas ezetimibe has no effect on plasma Lp(a) levels in a meta-analysis of 10 RCTs (Table 1) [20]. Despite the small increase in Lp(a) levels, prescription of statins reduces ASCVD risk, even in high Lp(a) patients. The only approved agents which result in significant plasma Lp(a) lowering are proprotein convertase subtilisin kexin type 9 (PCSK9) inhibitors (Table 1), although this is not a recognized indication for prescription. PCSK9 inhibition with evolocumab and alirocumab resulted in a median 27% reduction in Lp(a) levels in the FOURIER and ODYSSEY Outcomes trials, respectively [21,22]. However, in the FOURIER trial, the relative reduction was only 16% in the top quartile of Lp(a) levels compared to 28% in the other quartiles. Therefore, it seems that the relative reduction of Lp(a) is dependent on the baseline Lp(a) and thus relatively smaller in patients with higher Lp(a) levels. In two posthoc analyses from the FOURIER and ODYSSEY OUTCOMES trials, it was shown that the absolute Lp(a) reduction achieved with PCSK9 inhibitors was an independent predictor of reduction in MACE [23,24]. Thus, patients with the highest baseline Lp(a) levels and highest absolute Lp(a) reductions achieved the greatest clinical benefit with PCSK9 inhibition. Inclisiran also showed dose-dependent reductions of Lp(a) up to 26% [25]. Lastly, in patients with residual high ASCVD risk and high Lp(a) levels despite maximally tolerated lipid lowering therapy, lipid apheresis is approved in the United States for Lp(a) lowering and has shown to lower Lp(a), albeit transiently, by 63% postapheresis compared to preapheresis values [26].

**Table 1:** Properties of approved therapies and therapies in clinical trials on Lp(a) plasma levels

Name	Drug type	Drug target	Route of administration	Dosing frequency	Phase	Effect on Lp(a) levels
Statins	Competitive inhibitor	HMGCR	Oral	Daily	Approved	+10 to 20% (varying by type of statin) [19]
Ezetimibe	Cholesterol absorption inhibitor	NPC1L1	Oral	Daily	Approved	No effect [20]
Lipid apheresis	Apheresis	NA	NA	Weekly	Approved in USA	–63% postapheresis [26]
PCSK9i antibodies	Monoclonal antibody	PCSK9	Subcutaneous	Every 2 weeks	Approved	–27% [21,22]
Inclisiran	Small interfering RNA	PCSK9	Subcutaneous	Twice yearly	Approved	–19% to –26% [35]
Pelacarsen	GalNAc-conjugated antisense oligonucleotide	*LPA* mRNA	Subcutaneous	Once monthly	Phase 3	–80% [29^▪▪^]
Olpasiran	GalNAc-conjugated siRNA	*LPA* mRNA	Subcutaneous	Every 3 months	Phase 2	Up to –90% [31^▪^]

GalNAc, *N*-acetylgalactosamine; HMGCR, 3-hydroxy-3-methylglutaryl coenzyme reductase; Lp(a), lipoprotein(a); NPC1L1, Niemann-Pick-like protein 1C1; PCSK9i, proprotein convertase subtilisin kexin type 9 inhibiting; siRNA, small interfering RNA.

### How much lipoprotein(a) lowering is needed?

Evidence from Mendelian randomization by Burgess *et al.*[5] has suggested that the reductions in Lp(a) achieved with PCSK9 inhibition modeled by loss-of-function variants are not enough to achieve a significant reduction in cardiovascular events. Compared to LDL-C lowering, where 1 mmol/l reduction results in a 21–23% reduction in ASCVD risk, much larger absolute Lp(a) reductions are needed to achieve the same ASCVD benefit. In an analysis in almost 200 000 patients from 48 studies, Burgess *et al.*[5] estimated that a 101.5 mg/dl reduction in Lp(a) is needed to achieve the same ASCVD benefit as achieved with 1 mmol/l (38.7 mg/dl) LDL-C lowering. However, a more recent study from the Copenhagen General Population study with 58 527 secondary prevention individuals, showed that a 50 mg/dl reduction in Lp(a) for 5 years may already result in a 20% reduction in ASCVD events [27^▪^]. Whether this difference is based on the higher risk population or has other causes remains to be established. Nevertheless, these absolute reductions are much larger than what can be achieved with PCSK9 inhibition, supporting the need for specific and potent Lp(a) lowering strategies.

### Antisense oligonucleotide: pelacarsen

Pelacarsen is an antisense oligonucleotide covalently bound to an *N*-acetylgalactosamine (GalNAc_3_) to ensure specific uptake by the asialoglycoprotein receptor on hepatocytes [28]. It is administered once-monthly through a subcutaneous injection. Its phase 1/2a trial in healthy volunteers showed potent Lp(a) reductions up to 92% without signs of adverse events. The dose-ranging trial conducted in 286 patients with ASCVD history demonstrated a mean 80% reduction of Lp(a) at the highest dose, while 98% of patients reached the desirable level of 50 mg/dl (Table 1) [29^▪▪^,30^▪^]. Again, there were no major safety issues but pelacarsen was associated with more injection-site reactions compared to placebo. The ongoing phase 3 trial will investigate cardiovascular endpoint efficacy in 7680 patients with ASCVD and is expected to finish in 2024 (NCT04023552).

### Small interfering RNA: olpasiran

Olpasiran is a small interfering (si)RNA which reduces Lp(a) production through targeting of mRNA transcribed from the *LPA* gene [31^▪^]. It is administered every 3 months via a subcutaneous injection. A phase 1 study of olpasiran in healthy volunteers showed Lp(a) reductions of more than 90%, without signs of major safety concerns and persisting for at least 3 months (Table 1) [31^▪^]. The phase 2 dose ranging trial of olpasiran in patients with established ASCVD has finished recruiting and is expected to finish in 2023 (NCT04270760).

## CLINICAL CONSEQUENCES: ROUTINE LIPOPROTEIN(a) MEASUREMENT

There are several considerations supporting the routine measurement of Lp(a) in clinical practice.

First, individuals with extremely elevated Lp(a) (above the ^99th^ percentile; >180 mg/dl) have an Familial Hypercholesterolemia (FH)-like risk of ASCVD [30^▪^]. Even in patients at low risk of ASCVD according to traditional ASCVD risk scores, very high Lp(a) levels above 180 mg/dl can result in markedly increased ASCVD risk. In primary prevention, every 50 mg/dl Lp(a) increase translates to a hazard ratio of 1.16 for CVD mortality alone [16]. In fact, it was shown that incorporation of Lp(a) into the Systematic COronary Risk Evaluation (SCORE) risk algorithm in patients with Lp(a) >^99th^ percentile led to a 31% reclassification in primary prevention patients [32]. In secondary prevention, 63% of patients with very high Lp(a) were reclassified to a higher risk category of the Second Manifestations of ARTerial diseases (SMART) score [32].

Second, in patients with modestly elevated Lp(a) levels above 50 mg/dl but below 180 mg/dl at intermediate or high risk, Lp(a) can also lead to a significant risk increase [hazard ratio for myocardial infarction: 2.0, 95% CI (1.5–2.7)] [14]. In these patients, Lp(a) measurement could lead to changes of therapeutic regimens. In the absence of approved Lp(a) lowering therapies, further intensification of LDL-C lowering can be recommended to reduce residual ASCVD risk.

Lastly, Lp(a) is important for quantification of ‘true’ plasma LDL-C levels. Since Lp(a) mass largely aligns with LDL mass, both measured LDL-C and Friedewald-calculated LDL-C also contain Lp(a)-cholesterol. Therefore, ‘true’ LDL-C is generally overestimated, especially in high Lp(a) patients. To correct LDL-C for Lp(a)-C, the cholesterol part of Lp(a) mass should be subtracted from total LDL-C [33]. Based on recent insights from a novel direct Lp(a)-C quantification assay, it is estimated that 17.3% of total Lp(a) mass consists of cholesterol [9^▪^]. The quantification of ‘true’ LDL-C levels and thus Lp(a) measurement is important for two reasons: first, for therapeutic decisions regarding further LDL-C lowering. In patients with very high Lp(a) and relatively low LDL-C, LDL-C lowering will have no or limited effect on measured LDL-C, and could result in apparent ‘statin-refractory’ patients. Second, it is also important in FH diagnosis. When LDL-C is corrected for Lp(a)-C in patients suspected of FH, up to 23% of patients are no longer suspected of FH according to the Dutch Lipid Clinic Network (DLCN)-criteria [34]. Thus, Lp(a) measurement in these patients can lead to a considerable reduction in unnecessary genetic sequencing costs.

Given these considerations, the 2019 European Society of Cardiology (ESC)/European Atherosclerosis (EAS) advised to measure Lp(a) at least once in every adult [30^▪^]. Since specific Lp(a) lowering therapies are still in clinical trials, management of high Lp(a) levels should focus on reducing residual ASCVD risk resulting from other risk factors. Most importantly, the ‘true’ LDL-C should be further reduced with statins and ezetimibe, where necessary supplemented by PCSK9 inhibitors (which also provide a modest Lp(a) reduction). Additionally, attention should be given to other ASCVD risk factors such as hypertension and improving lifestyle factors to minimize ASCVD risk from other factors than Lp(a).

## FUTURE PERSPECTIVES: OPTIMIZATION OF ATHEROSCLEROTIC CARDIOVASCULAR DISEASE RISK SCORES

As discussed, both very high Lp(a) as well as modestly elevated Lp(a) may have significant consequences for ASCVD risk stratification. Considering Lp(a) is a likely causal and independent risk factor for ASCVD, novel ASCVD risk scores should implement Lp(a). As was shown previously, hazard ratios from observational studies can easily be implemented into established risk scores such as SCORE and SMART [32]. When validated in external cohort data, this should be the first step. Since Lp(a) is routinely measured in an increasing number of hospitals and countries, new risk scores comprising Lp(a), which should be based on large-sized cohorts can and should be developed in the near future.

## CONCLUSION

Lp(a) should be measured in every patient at least once, given the potential consequences for ASCVD risk stratification, especially to identify adult patients with low ASCVD risk according to traditional ASCVD risk scores but with unknown very high Lp(a) levels. The next step will be to incorporate Lp(a) into daily used ASCVD risk scores. If approved, Lp(a) lowering therapies can be prescribed in high Lp(a) adult patients. Until then, the therapeutic strategy should focus on reducing residual lipid driven risk by further reducing LDL-C and other CVD risk factors.

## Acknowledgements

*None.*

### Financial support and sponsorship

*None.*

### Conflicts of interest

*N.S.N. is co-founder of Lipid Tools. P.M. reports grants and personal fees from Regeneron, Amgen, Esperion, Kaneka, Stage II Innovations/Renew, grants from Novartis, Ionis Pharmaceuticals, FH Foundation, GB Life Sciences, Aegerion and personal fees from Amarin. ESGS reports advisory board/lecturing fees paid to the institution of ESGS by Amgen, Sanofi, Regeneron, Esperion, Novo-Nordisk, Esperion, IONIS.*

## REFERENCES AND RECOMMENDED READING

Papers of particular interest, published within the annual period of review, have been highlighted as:

▪ of special interest
▪▪ of outstanding interest
